# Protective Effect of *Matricaria chamomilla* Ethanolic Extract on Hippocampal Neuron Damage in Rats Exposed to Formaldehyde

**DOI:** 10.1155/2018/6414317

**Published:** 2018-08-14

**Authors:** Zahra Sayyar, Alireza Yazdinezhad, Maryam Hassan, Iraj Jafari Anarkooli

**Affiliations:** ^1^Department of Anatomy, Faculty of Medicine, Zanjan University of Medical Sciences, Zanjan, Iran; ^2^Department of Pharmacognosy, School of Pharmacy, Zanjan University of Medical Sciences, Zanjan, Iran

## Abstract

Formaldehyde, as a frequently used compound in many applications, crosses the blood-brain barrier and leads to hippocampal cell death and memory impairment. This study investigates the effects of ethanolic extract of *Matricaria chamomilla* (MC) on passive avoidance learning induced by damaged hippocampal cells and evaluates the antioxidant traits of MC. The male Wistar rats were divided into six (6 in each) groups: control (10 mg/kg normal saline), 200 (200 mg/kg MC extract), 500 (500 mg/kg MC extract), F (10 mg/kg formaldehyde), F200 (10 mg/kg formaldehyde and 200 mg/kg MC extract), and F500 (10 mg/kg formaldehyde and 500 mg/kg MC extract). Shuttle box assay was used for evaluation of passive avoidance learning. The apoptosis rate of hippocampal tissue, malondialdehyde (MDA) free radicals, and total antioxidant capacity was evaluated to determine the positive effect of the ethanolic extract of MC. We found that the ethanolic extract of MC reduced the cell death, time spent in a dark room, and MDA free radicals in the hippocampus, leading to increased total antioxidant capacity in this region. In conclusion, the ethanolic extract of MC could ameliorate formaldehyde-induced memory damage through decreasing cell death and MDA activity of the hippocampal region and increasing total antioxidant capacity.

## 1. Introduction

Formaldehyde (HCHO), the simplest form of aldehyde family, is a gas colorless at room temperature and a common pollutant that can cross the blood-brain barrier. Environmental pollutions, diet, aging, and genetic factors can influence the accumulation of HCHO in the brain [1]. In addition to oxidative stress production, formaldehyde can affect DNA methylation and hydrogen sulfide production, which are important factors in forming and increasing long-time memory [2]. Formaldehyde plays an important role in oxidative stress [3, 4]. Since this compound can easily pass through the blood-brain barrier, it leads to death and damage to the cells of the brain regions, especially hippocampus cells [5, 6]. In this regard, the loss of cells in the hippocampus region results in memory loss and cognitive dysfunctions [7]. Formaldehyde impairs the oxidant-antioxidant balance because it induces oxidative stress, forms reactive oxygen species (ROS), and increases lipid peroxidation in damaged tissues. Overproduction of these species leads to increased toxicity through oxidative damage to cell compartments such as the DNA, protein, and lipids and induces biological and pathological impacts such as mitogenesis, differentiation, mutagenesis, and cell death [8]. Formaldehyde can lead to cellular apoptosis under in vitro condition [9]. Apoptosis is a mechanism of programmed cell death that is essential for maintaining cell populations within tissues. Malregulation of apoptosis is the principal cause of many diseases. Neuroplasticity of the hippocampus makes it the most susceptible region of the brain to oxidative stress [10]. Oxidative stress exerts its deleterious effects through free radical production in several illnesses such as Alzheimer's and Parkinson's diseases and cancer. The deleterious effects of free radicals, which destroy the cell membrane, are through lipid peroxidation. Antioxidants are most important defensive factors against free radicals. Antioxidants exert their physiologic functions through free radical scavenging, specifically superoxide and hydroxyl anions [11].

Medicinal herbs contain precious compounds that can increase the antioxidant capacity of blood plasma. Antioxidants are found in two forms: natural and synthetic. Despite the synthetic form of antioxidants, the natural form contains no side effects; therefore, natural antioxidants can be considered as an effective solution in reducing free radical effects [12]. Beside the antioxidant effects, *Matricaria chamomilla* (MC) contains the highest anti-inflammatory impact among medicinal herbs. MC is an annual plant of the family Asteraceae that is used as a painkiller, antispasmodic, and antibacterial agent in traditional medicine. It is used to cure skin diseases such as psoriasis, eczema, and bronchitis; the common cold; coughing; fever; wounds; and gastrointestinal problems. MC is rich in flavonoids, which are effective antioxidants in neutralizing free radicals [13]. Previous studies have shown neuroprotective effects of MC against ischemia and fluoride [14]. In recent years, researchers have focused on protective antioxidant effects of antioxidants against brain damage induced by formaldehyde. Since herbs are a source of natural antioxidants and do not accompany the side effects of artificial antioxidants, we conducted this study to investigate the protective effects of ethanolic MC extract on rats' hippocampal damage induced by formaldehyde. Furthermore, we evaluated the effects of ethanolic MC extract on passive avoidance learning, as well.

## 2. Materials and Methods

### 2.1. Preparation of *Matricaria chamomilla* Extract

*Matricaria chamomilla* was collected from Tarom County of Zanjan Province during the blooming season. The collected samples were confirmed in the School of Pharmacy of Zanjan University of Medical Sciences with the herbarium code of ZUMS-4112. The collected samples were dried in darkness and at room temperature and powdered by the electric grinder. The powder was collected in a decanter and mixed with 70% ethanol. Extracts were collected after 24, 48, and 72 h and filtered. Then, using the rotary evaporator (60°C and 3000 rpm), the solvent was removed, and the concentrated solution was kept under appropriate conditions (darkness and under the laminate hood) to obtain a powder. Gas chromatography/mass spectrometry (GC/MS) analysis of *Matricaria chamomilla* extract was performed using GC-MSD Agilent GC; a gas chromatography was attached to a mass spectrometer equipped with an HP5 od 0.25 *μ*m × 30 m column. The desired concentration of extract was prepared by weighing the powder and mixing it with a physiological serum to prepare a thin suspension, which was injected intraperitoneally to rats [15].

### 2.2. Evaluation of Antioxidant Activity by 2,2-Diphenyl-1-picrylhydrazyl (DPPH)

**Figure 1:**
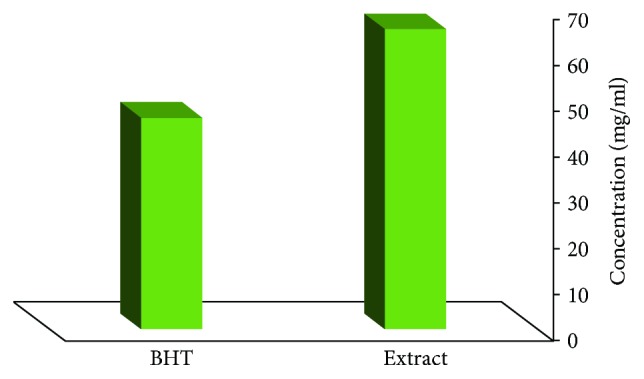
Comparing IC50 of *Matricaria chamomilla* Extract with BHT Antioxidant.

To investigate the antioxidant activity of MC, a stable radical compound (DPPH) was used as an identifier, and hydroxy toluidine blue (HTB) as a common antioxidant was used as positive control. Concentrations of 500, 250, 125, 62.5, 31.25, 15.62, 7.81, and 3.905 mg/ml of HBT were prepared. To prepare the desired concentration of the extract, doses of 461, 230.5, 115.3, 57.6, 28.8, 14.41, 7.21, and 3.6 mg/ml of extract were prepared by adding methanol. About 500 *μ*l of DPPH (0.004%) and 500 *μ*l of various doses were added to 16 Falcon tubes. The tubes were vortexed for 15 min and kept for 30 min in darkness. At last, spectrophotometry of the samples was performed at 517 nm wavelength, where methanol was considered as blank. The procedure was repeated three times. The solution containing DPPH turns color from dark blue/purple to yellow. Light absorbance at 517 nm wavelength was measured by the following formula [16]:

(1) $$\begin{array}{c} I\%=\left(1-\frac{A_{test}}{A_{control}}\right)\times 100, \end{array}$$

where ACONTROL is the amount of light absorbance in the control group (DPPH solution) and ATEST denotes the amount of light absorbance at different doses of MC. Finally, the concentration of MC, which suppresses 50% of radicals, was measured by a diagram (Figure 1).

### 2.3. Phenolic Compound Identification

To measure the phenolic compounds, Folin-Ciocalteu reagent and gallic acid were used as standard [17]. About 1 ml of MC extract (100 *μ*g/ml in methanol) was mixed with 5 ml of Folin-Ciocalteu. After 3 min, 10 ml of sodium bicarbonate 2% was added and mixed for 15 min. Then, it was kept for 30 min in darkness, followed by performing spectrophotometry at 760 nm wavelength. The same procedure was performed for all the standard gallic acid solutions in distilled water (0–200 *μ*g/ml) using the standard diagram. The concentration of phenolic compounds was determined as mg of gallic acid per gram of dried ethanolic extract.

### 2.4. Anthocyanin Compound Identification

The method of anthocyanin compound identification is based on UV absorbance of anthocyanin molecule, which is accompanied by pH alteration of the solution [18]. First, 2 ml of MC extract (100 *μ*g/ml ethanol) was mixed with 23 ml of buffer solution (pH = 1; a mixture of 125 ml of 0.2 M potassium chloride and 375 ml of 0.2 M hydrochloric acid). Then, another 2 ml of MC extract was mixed with 23 ml of buffer solution (pH = 4.5; a mixture of 200 ml of 1 M sodium acetate, 120 ml of 1 M hydrochloric acid, and 160 ml of distilled water). Finally, the UV absorbance of the samples was read at 510 nm wavelength. The concentration of anthocyanin was measured by the following equation:

(2) $$\begin{array}{c} C\left(mg/l\right)=\frac{\left(Abs\;pH\;1-Abs\;pH\;4.5\right)\times 484.82\times 1000}{24825\times DF}, \end{array}$$

where *C* indicates concentration of anthocyanin compounds of MC extract based on cyanidin 3-glucoside; Abs pH = 1 indicates absorbance of the sample with pH = 1; Abs pH = 4.5 indicates absorbance of the sample with pH = 4.5; 484.82 and 24825 are, respectively, molecular mass and molar absorbance coefficient of cyanidin 3-glucoside at 510 wavelengths in the buffer solution; and DF is the dilution factor.

### 2.5. Flavonoid Compound Identification

To identify flavonoid compounds, aluminum chloride (AlCl_3_) spectrophotometry was used [19]. For this purpose, 4 ml of distilled water and 0.3 ml of NaNo_2_ 5% were added to 1 ml of MC extract (100 *μ*g/ml of ethanol) and shaken for 5 min. Then, 0.3 ml of AlCl_3_ 10% and 2 ml of 1 M sodium hydroxide were added, and the final volume of 10 ml was achieved by adding distilled water. Following 30 min of leaving it at room temperature, the absorbance of the samples was measured at 415 nm wavelength. Calibration curve of the coerestin solution was prepared at 0–50 *μ*g/ml methanol, and the results were reported as mg equal to coerestin per gram of dried ethanolic MC extract.

### 2.6. Animals

The adult male Wistar rats weighing 200–250 g (48 rats divided into six different groups and a normal control group) were housed under controlled standard conditions (a 12-hour light-dark cycle, 21-22°C room temperature, and 45–55% humidity). The rats were given ad libitum access to food and water. The animals were divided into 6 groups: (C) control group (received 10 mg/kg normal saline), 200 (received 200 mg/kg MC extract), 500 (received 500 mg/kg MC extract), F (received 10 mg/kg formaldehyde), F200 (received 10 mg/kg formaldehyde and 200 mg/kg MC extract), and F500 (received 10 mg/kg formaldehyde and 500 mg/kg MC extract). Initial weights of all the animals were measured, and the animals were marked to receive the specific dose of formaldehyde and MC extract based on their weights. Treatment course was 30 days in which formaldehyde and MC extract were injected daily and intraperitoneally. Before injection of MC extracts, 200 mg/kg and 500 mg/kg MC extracts were solved in normal saline. For groups F200 and F500, after 1 h of formaldehyde injection, the specific doses were injected. A vehicle group receiving 0.9% normal saline daily and intraperitoneally was designed as well.

### 2.7. Behavioral Test

The behavioral assessment was evaluated using the shuttle box unit. The structure of the unit and procedure was described previously in [20]. The step-through passive avoidance apparatus consisted of a lighted chamber (20 × 20 × 30 cm^3^) made of translucent plastic and a dark box with walls made of dark nontransparent plastic (20 × 20 × 30 cm^3^). The lower surface of the rooms of both chambers was made of stainless steel rods (3 mm diameter) spaced 1 cm apart. The floor of the dark chamber could be electrified using a shock generator. A rectangular opening (6 × 8 cm^2^) was located between the two chambers that could be closed by a nontransparent guillotine door. To train the animals, they spontaneously were entered into the dark chamber, the guillotine door was lowered, and a mild electrical shock (0.5 mA) was applied for 3 sec, after which the rat was returned to its home cage. Then, after 2 min, the procedure was repeated. The rat received a foot shock each time when it reentered the dark compartment and had placed all its four paws in the dark compartment. The training was terminated when the rat remained in the light compartment for 120 consecutive seconds. The number of trials (entries into the dark chamber) was recorded. After 30 days, passive avoidance learning of rats was evaluated by using the shuttle box. In this test, four criteria of passive avoidance learning and memory were evaluated: (1) step-through latency in the acquisition trial, (2) the number of trials, (3) step-through latency in the retention trial, and (4) time spent in dark compartments.

### 2.8. Histopathological Evaluation

The rats were sacrificed under deep ketamine and xylazine anesthesia. Their skull was dissected through midline to remove their brain and put on ice. The meninges were carefully removed, and the hippocampi were meticulously dissected and removed. One of the hippocampi was stored for histopathological assays and the other for total antioxidant capacity and MDA activity. For histopathological studies, tissues were fixed at formaldehyde 10% for 3 days. Following the fixation, the samples were processed with tissue processor for 25 h. Then, tissues were embedded into paraffin blocks. Microtomy was performed with a 5 *μ*m slice thickness. Before performing staining assays, slides containing tissues were incubated at 80°C for 1 h. Then, for cell death (apoptosis and necrosis) evaluation, two methods of staining were used: terminal deoxynucleotidyl transferase (TdT) enzyme-mediated dUTP nick end labeling (TUNEL), in which apoptotic cells are stained brown and normal cells are stained blue, and acridine orange staining, in which dead cells are stained orange and normal cells are stained black. To investigate the amount of cell death, 5 samples were selected. Then, 5 slices from each sample and 3 regions (Cornu Ammonis 1 (CA1), CA3, and dentate gyrus) in each slice were investigated. The number of dead cells and the total number of cells were counted and analyzed.

### 2.9. Total Antioxidant Capacity (TAC) Evaluation of the Hippocampus

TAC evaluation of the hippocampi was performed by using ferric reducing ability of plasma (FRAP) assay. This assay is based on iron (III) to iron (II) ion reduction in tripyridyl-s-triazine (TPTZ) complex. TPTZ complex is a coloring ligand that produces a deep blue color. To prepare the FRAP solution, 3 compounds of acetate (300 mM), iron (III) chloride (20 mM), and TPTZ (10 mM) were mixed by the ratio of 10 : 1 : 1. The standard curve of FRAP was diagrammed by various concentrations (0, 125, 250, 500, 750, and 1000 *μ*M) of iron (II) phosphate. After addition of 1.5 ml of FRAP agent to 50 *μ*l of homogenized samples of the hippocampi and leaving it for 10 min, light absorbance at 593 nm wavelength was read. By the use of standard curve, data were reported as *μ*M of iron (II) per g of the sample [21].

### 2.10. MDA Free Radical Activity of the Hippocampus

Thiobarbituric acid reactive substances (TBARS) were used for lipid peroxidation monitoring. For this purpose, 0.1 ml of homogenized hippocampus sample was mixed with 0.5 ml of trichloroacetic acid (TCA) 20% (20 g TCA in 100 ml distilled water) and kept at 30°C for 10 min. Then, it was centrifuged at 4°C and 7500 rpm for 10 min. The supernatant was removed, and 0.5 ml of 0.05 M sulfuric acid (270 *μ*l sulfuric acid in 100 ml distilled water) and 0.4 ml TBA (0.2%) was added to the pellet and left at 100°C hot water bath for 30 min. After cooling the samples, 0.8 ml n-butanol was added and centrifuged at 10,000 rpm for 10 min, and then the supernatant was read by spectrophotometer at 532 nm wavelength [22].

### 2.11. Statistical Analysis

Data were presented as a mean percentage ± SEM and analyzed by one-way ANOVA followed by Tukey's post hoc multiple group comparison test. The difference between the groups was considered statistically significant at *P* < 0.05.

## 3. Results

### 3.1. GC/MS Analysis

The components present in the *Matricaria chamomilla* extract were identified by GC/MS analyses. The active principles with their retention time, molecular concentration (percent) in the hydroethanolic extract, and CAS number are presented in Table 1.

### 3.2. Standardization of Ethanolic Extract of MC

According to Table 2, phenolic compounds in the dried extract sample were 284.6 ± 16 mg/g. The concentration of ethanolic extract of MC, which leads to a 50% inhibition of the oxidative activity of DPPH radicals (IC_50_), is 65.4 *μ*g/ml while for butylated hydroxytoluene (BHT), the concentration is 46.23 μg/ml. Although the efficiency of ethanolic extract in scavenging DPPH radicals is lower than that of synthetic antioxidant of BHT, the ethanolic extract of MC is of considerable antioxidant activity and can reduce the amount of DPPH free radicals. According to Table 2, the total anthocyanin compounds based on cyanidin 3-glucoside molecule were 0.86 ± 0.17 mg/g dried MC extract. Detecting flavonoid compounds by the method of AlCl_3_ spectrophotometry revealed 211.2 ± 0.13 coerestin in each gram of dried MC extract.

### 3.3. Behavioral Test Result: Passive Avoidance Learning

**Figure 2:**
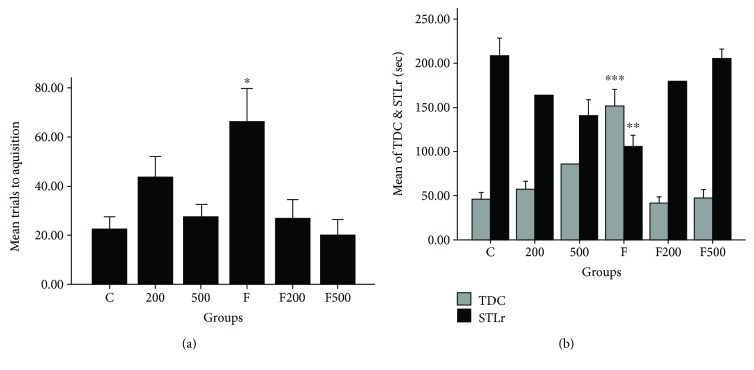
Impact of MC extract on a number of trials between the two compartments in the passive avoidance learning in study groups (a). Impact of MC extract on step-through latency in the test time and total time spent in a dark room in the passive avoidance learning in the study group (b). C: control group; F: formaldehyde-treated group; 200: 200 mg/kg MC-treated group; 500: 500 mg/kg MC-treated group; F200: formaldehyde plus 200 mg/kg MC-treated group; F500: formaldehyde plus 500 mg/kg MC-treated group. ^∗^*P* < 0.05 F groups versus other study groups; ^∗∗^*P* < 0.01 F group versus other study groups; ^∗∗∗^*P* < 0.001 F group versus other study groups. Data are reported as mean ± SEM.

To ensure that there is no sensory/motor dysfunction in animals, the initial latency was measured and compared. Comparison between groups showed no significant step-through latency in the acquisition trial (STLa). The memory assessment test, 24 h following the training, indicated that the number of the trial for the acquisition of the animal between dark and light chambers is statistically significant between groups (*P* < 0.05). A comparison between the studied groups showed that there is a statistically significant difference between groups F and C (*P* < 0.05), suggesting that formaldehyde toxicity leads to increased number of the accusation trials of the animal between chambers. Also, a significant difference was identified between groups F and F200 (*P* < 0.05) and F500 (*P* < 0.05). There is also a significant difference between groups C and 200 (*P* < 0.05) and 500 (*P* < 0.05), suggesting the positive effects of ethanolic extract of MC in preventing memory damage in rats (Figure 2(a)). Twenty-four hours after the training, memory evaluation indicated that step-through latency (STLr) in group F is decreased significantly compared to group C (*P* < 0.001), indicating the decreased learning caused by formaldehyde toxicity. Also, in group F compared to groups F200, F500, 200, and 500, the rate of latency was decreased considerably (Figure 2(b)). The total time spent in the dark compartment was extensively influenced by formaldehyde in comparison to group C (*P* < 0.01), in which the number of trials between two compartments and consequently the time spent in the dark compartment in group F were significantly increased compared to those of group C (*P* < 0.01). The number of trials between two compartments and the time spent in the dark compartment in F200, F500, 200, and 500 were significantly reduced in comparison to those of group F (Figure 2(b)).

### 3.4. Histopathological Analysis (TUNEL Assay)

**Figure 3:**
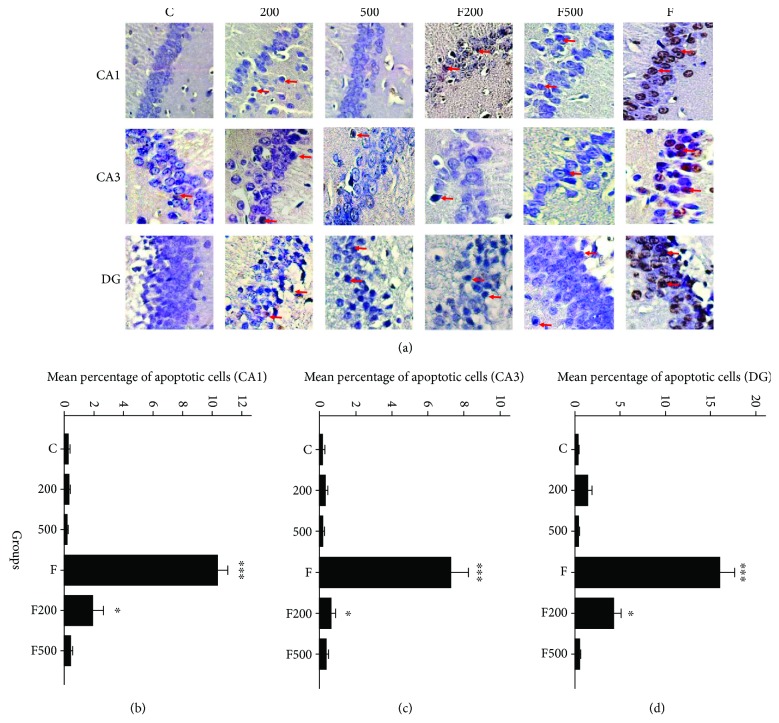
Micrographs of TUNEL assay of rats' hippocampi (CA1, CA3, and DG regions): C: control group; F: formaldehyde-treated group; 200: 200 mg/kg MC-treated group; 500: 500 mg/kg MC-treated group; F200: formaldehyde plus 200 mg/kg MC-treated group; F500: formaldehyde plus 500 mg/kg MC-treated group. Arrows indicate apoptotic cells; MC impact on decreasing number of apoptotic cells in rats' hippocampi (CA1, CA3, and DG regions); ^∗∗∗^*P* < 0.001 C, 200, 500, FM500 groups versus F group. ^∗^*P* < 0.05 F200 group versus F group.

The results indicate that there is a significant increase in TUNEL positivity between group F and other studied groups in the CA1, CA2 (*P* < 0.001), and DG (*P* < 0.05) regions. As can be seen, the MC extract has a neuroprotective effect in all the MC-treated groups. However, the difference in the number of dead cells in the F500 group is lower than that in the other experimental groups (Figure 3).

### 3.5. Histopathological Analysis (Acridine Orange Assay)

**Figure 4:**
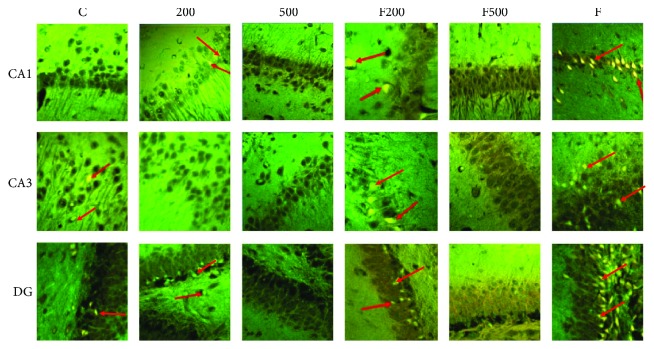
Micrographs of acridine orange assay of rats' hippocampi (CA1, CA3, and DG regions): C: control group; F: formaldehyde-treated group; 200: 200 mg/kg MC-treated group; 500: 500 mg/kg MC-treated group; F200: formaldehyde plus 200 mg/kg MC-treated group; F500: formaldehyde plus 500 mg/kg MC-treated group. Arrows indicate dead cells.

Evaluation of regions CA1 and CA3 shows a significant difference in the number of dead cells in group F compared to the other groups (*P* < 0.001), indicating the positive impact of MC extract on reducing cell death in the hippocampus. A significant increase in the number of dead cells in DG region in group F compared to other groups is also evident (*P* < 0.001). Although there is a significant difference between F and F500 group (*P* < 0.05), it is not as much as that of other groups (Figure 4, Table 3).

### 3.6. TAC Evaluation of the Hippocampus

**Figure 5:**
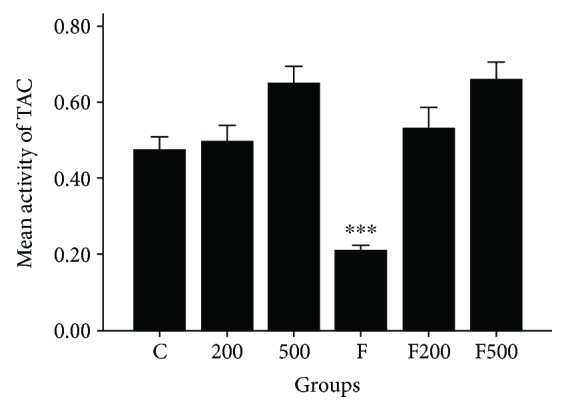
MC impact on TAC in rats' hippocampi. ^∗∗∗^*P* < 0.001 C, 200, 500, F200, and F500 groups versus F group. C: control group; F: formaldehyde-treated group; 200: 200 mg/kg MC-treated group; 500: 500 mg/kg MC-treated group; F200: formaldehyde plus 200 mg/kg MC-treated group; F500: formaldehyde plus 500 mg/kg MC-treated group.

Iron concentration was significantly decreased in group F in comparison to group C. In the FM500 group, there is a significant increase in iron concentration in comparison to the control group (*P* < 0.001). A considerable increase in iron concentration in the MC500 group was evident as well (Figure 5).

### 3.7. MDA Free Radical Evaluation in the Hippocampus

**Figure 6:**
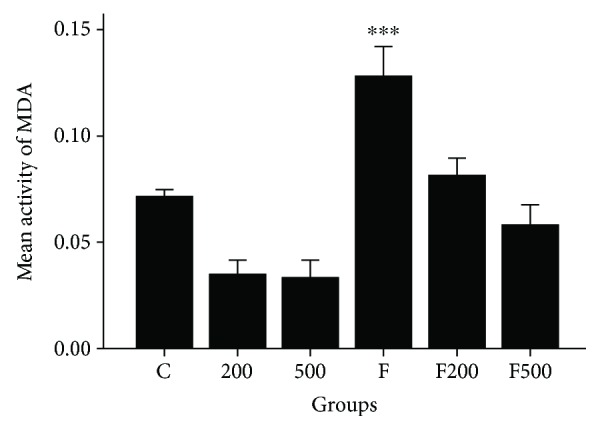
MC impact on MDA free radicals in rats' hippocampi. ^∗∗∗^*P* < 0.001 200, 500 C, F200, and F500 groups in comparison to group F. C: control group; F: formaldehyde-treated group; 200: 200 mg/kg MC-treated group; 500: 500 mg/kg MC-treated group; F200: formaldehyde plus 200 mg/kg MC-treated group; F500: formaldehyde plus 500 mg/kg MC-treated group.

A statistically significant difference was evident between groups C and F (*P* < 0.001). Moreover, there was a statistically significant difference between group F and groups FM200, FM500, MC200, and MC500 (*P* < 0.001, Figure 6).

## 4. Discussion

The results of this study show that intraperitoneal injection of 10 mg/kg of formaldehyde leads to increased cell death in the hippocampi of rats. According to Zararsiz et al., increased apoptosis in the prefrontal cortex of rats receiving formaldehyde is due to the transportation of cytochrome c from the mitochondrial membrane into the cytoplasm and increased activity of Bax protein. The results showed that cell (death) induced by formaldehyde toxicity is due to the enhanced Bax proteins activity. Formaldehyde toxicity and the oxidative stress generated in the brain tissue were reported in two separate papers by Zararsiz et al. Both studies evaluated the oxidative stress markers (SOD, GSH, and MDA) in rats injected with formaldehyde and under the treatment of melatonin and omega-3 antioxidants. The results of these studies were in agreement with those of the previous studies that reported the apoptotic cell death induced by increased ROS and oxidative stress, confirmed by the increased peroxidation index and decreased antioxidant capacity in the brain tissue. Furthermore, in these studies, signals from external mitochondrial membrane due to increased proapoptotic and decreased antiapoptotic proteins are reported as causes of cell death [2, 23].

Arici et al. treated rabbits with formaldehyde and observed a decrease in Bcl-2 expression while an increase in caspase-3 and Bax proteins. The resulted apoptotic cell death was due to increased ROS and imbalance of oxidant-antioxidant system. Furthermore, they reported that increased activity of aldehyde oxidase, peroxidase, and xanthine oxidase in endoplasmic reticulum causes increased ROS level. Formaldehyde is considered as a substrate for P-450. Increased activity of the mentioned enzymes leads to some damage to the cell membrane lipids, proteins, and nucleic acids. Since the nervous system is rich in unsaturated fatty acids, increased activity of these enzymes and ROS simply leads to lipid peroxidation, and consequent damage to the nervous system is inevitable. According to this study and other similar works, formaldehyde-induced apoptosis in cells occurs through oxidative stress and internal mitochondrial pathway [24].

The results of the present study show that injection of 10 mg/kg formaldehyde can lead to lipid peroxidation in the hippocampus and increase reactive oxygen species. The ethanolic extract of MC decreases MDA level considerably. According to identical studies, increased MDA level can be associated with glutathione resulted from formaldehyde aggregation, increased free radicals, and increased cell membrane lipid peroxidation. In other words, formaldehyde causes electron transport chain impairment, which leads to increased reactive oxygen species, lipid peroxidation, and decreased glutathione. These factors cause decreased ATP synthesis and mitochondrial ATP/ADP ratio, which lead to a decreased mitochondrial membrane potential, cytochrome c release, cellular apoptosis, and necrosis. Several studies with the purpose of preventing oxidative stress-induced brain damage have been conducted on antioxidants that can cross blood-brain barrier (BBB) [25]. Since antioxidant characteristics of MC with the ability to cross BBB in the nervous system under formaldehyde treatment have not been investigated yet, we aimed to investigate antioxidant effects of MC on the hippocampus under formaldehyde treatment.

Our results show that ethanolic MC extract can prevent apoptosis in the hippocampus. The evaluation of MDA free radicals indicated that MC extract extensively prevented lipid peroxidation as well. Ranpariya et al. investigated the effect of methanolic MC extract on lipid peroxidation inhibition in aluminum fluoride-induced oxidative stress and reported that the lipid peroxidation inhibition is caused by chamazulene found in MC. Rabiei et al. induced memory loss in rats by the use of scopolamine, a muscarinic acetylcholine receptor blocker, followed by injection of MC extract as a treatment, and investigated oxidative stress and rate of memory loss in treated rats. They reported that MC extract could considerably improve learning. Moreover, it has been reported that improvement in motor impairment is caused by MC antioxidant characteristics and phenolic compounds since there was a considerable decrease in MDA free radicals in the rat brain tissue and an increase in glutathione and catalase enzyme levels [26].

Mechanisms suggested for protective effects of medicinal herbs consist of decreased oxidative and nitrosative stress; decreased lipid peroxidation, preventing DNA fragmentation; decreased microglia and astrocyte activity; inhibition of apoptotic protein expression; increased mitochondrial gene expression; decreased leukotrienes, prostaglandins, and thromboxane; increased antiapoptotic protein expression; and decreased inflammatory mediator expression [14]. Our results are in line with those of Abdanipour et al., who reported a considerable decrease in the apoptosis of hippocampal cells in an in vitro condition under oxidative stress and MC treatment. In their study, the self-proliferative activity of cells is assumed to be associated with flavonoid compounds such as apigenin, coumarin, coerestin, and alpha-bisabolol. Furthermore, their TUNEL assay results imply the antiapoptotic MC activity on damaged cells in an oxidative stress condition [27]. Several studies have reported the radical scavenging and antioxidant effects of *Matricaria chamomilla* and neuroprotective effect of *Matricaria chamomilla* [3, 28, 29].

Flavonoid is one of the essential compounds in medicinal herbs. Flavonoid-rich diets containing extracts such as blueberry, green tea, and saffron exert positive effect of Alzheimer's, Parkinson's, and epilepsy diseases, suggesting the positive impacts of flavonoids on memory. Flavonoid compounds cause vasculogenesis and neurogenesis in the hippocampus, which inhibit apoptosis induced by toxic species [30]. Antioxidant effects of flavonoids in neurodegenerative diseases, such as epilepsy, were investigated in [31]. However, following MC treatment, we observed a considerable increase in TAC in the hippocampus. In many of the previous studies on the impact of MC on other tissues, identical results were obtained. Previous studies show that herbs containing flavonoid compounds have stronger healing effects [32]. Mahmoodzadeh et al. reported increased TAC in tissues after MC extract treatment. They observed carbon tetrachloride poisoning induced in rats and then investigated the healing effects of MC extract. They also reported that the increased TAC is due to hydroxyl and phenolic compounds [33]. Wu et al. reported that the arrangement of hydroxyl groups in chemical compounds of MC is in such a way that makes it more prone to donate electron in comparison to other compounds containing hydroxyl group [34]. Therefore, the increased TAC in our study groups is caused by hydroxyl compounds found in the ethanolic extract of MC. Identification of phenolic compounds in MC extract confirms the existence of these precious compounds in MC (Table 2).

Our results show that following cell death, a learning decrease is seen in rats receiving formaldehyde. The rats receiving MC extract are better in learning compared to those that have not received the extract. Reviewing the related literature suggests that aggregated formaldehyde in the brain can induce its adverse effects on molecular coding process and memory storage through increased tau protein phosphorylation, cellular apoptosis, decreased DNA methylation, and decreased efficiency of the noradrenergic system [8]. The most important reason for the application of medicinal herbs as a treatment for memory loss and memory boosting is the polyphenolic and flavonoid compounds with antioxidant effects and precursors releasing acetylcholine, inhibitors of acetylcholinesterase, and compounds releasing dopamine transporters [35]. In many studies on effects of flavonoids on the nervous system, GABA receptors and ligands found in these compounds are mentioned as molecules that act like benzodiazepines. Therefore, in studies on MC, positive effects on seizure have been observed [36]. Shakeri et al. report flavonoids can decrease calcium ions that enter the cell and consequently control NMDA receptor activity; this is done by the decreased nitric oxide and calcium-dependent phospholipase synthesis [37]. Misane et al. induced memory loss in rats through injection of scopolamine and consequent oxidative stress. Then, they treated them with MC extract and studied their learning and memory through two methods; that is, Morris water maze and shuttle box. They reported that MC extract could improve learning and memory in the studied rats [38].

Memory boosting following MC consumption indicates its action mechanism in scavenging free radicals, inhibiting lipid peroxidation, and antiacetylcholinesterase effects [39]. Rabiei et al.'s study investigated the impact of MC extract on passive avoidance memory and learning of rats by shuttle box. According to their results, MC extract has boosting effects on rats' memory. They also reported important compounds such as camphor, p-cymene, carvacrol, terpinen 4-ol, and borneol in MC extract. According to Rabiei et al., these compounds are able to inhibit acetylcholinesterase enzyme [26].

Therefore, ethanolic MC extract can boost learning and memory through various mechanisms. One of these probable pathways is through inhibiting acetylcholinesterase enzyme and consequently the increased acetylcholine level in the brain and hippocampus. Another possible mechanism is the impact of MC on glutamate NMDA receptors. Previous studies have shown the high number and density of glutamate receptors in the hippocampus. These receptors induce a cascade of signals in the cells through increased inner cell calcium ions and increase synaptic activity of hippocampal cells and lead to long-term potentiation (LTP). Since there is a direct and indirect interference between NMDA and cholinergic system, MC can boost memory and learning through influencing LTP system [1].

Finally, according to the previous studies and the present study, formaldehyde-induced neurotoxicity can lead to cognitive and behavioral disorders in male rats through oxidative stress and increased apoptosis rate in the hippocampus. MC extract with antioxidant effects and important compounds can decrease side effects induced by oxidative stress and lead to improved learning and memory in rats.

## 5. Conclusion

According to the results of our study, the ethanolic extract of MC can possibly ameliorate formaldehyde-induced memory damage through decreasing cell death and MDA activity of the hippocampal region and increasing the total antioxidant capacity.

## Figures and Tables

**Table 1:** Components detected by gas chromatography/mass spectrometry (GC/MS) in the MC extract.

Row	Compound	*R*_t_	Percent	CAS
1	3-Fluorophenethylamine	34771	0.20	000404-70-6
2	1,2,2-Trimethylcyclopropylamine	7706	13.96	000000-00-0
3	Heptacosane $$ n-Heptacosane	320679	33.53	000593-49-7
4	7-Methoxy-2,3,4,5,6,7-hexahydro	233273	6.13	099659-20-8
5	Hex-5-enylamine	7716	4.48	034825-70-2
6	2,6,10,14,18,22-Tetracosahexaene	337963	16.71	007683-64-9
7	1,2-Benzenedicarboxylic acid	131213	5.99	000603-11-2
8	Phenol, 4-(2-aminoethyl)	32743	5.26	000051-67-2

**Table 2:** Antioxidant activity, phenolic and flavonoid compounds, and anthocyanin of MC.

Sample	Method used			
	Scavenging method of DPPH radical^d^	Flavonoid test^c^	Anthocyanin test^b^	Phenolic compound test^a^
Ethanolic extract	56.40 ± 1.45	211.20 ± 0.13	0.86 ± 0.17	284.60 ± 16
BHT	46.23 ± 0.23	—	—	—

^a^mg gallic acid per g of ethanolic extract of dried MC; ^b^mg cyanidin 3-glucoside per g of ethanolic extract of MC; ^c^mg coerestin per g of ethanolic extract of MC; ^d^IC_50_ (*μ*g/ml).

**Table 3:** MC impact on the number of dead cells in rats' hippocampi (CA1, CA3, and DG regions).

Group outcome	Mean ± SD					
	C	200	500	F	F200	F500
CA1 area	0.22 ± 0.15	0.31 ± 0.23^∗^	0.24 ± 0.21	5.72 ± 1.11^∗∗∗^	0.89 ± 0.36^∗^	0.25 ± 0.19
CA3 area	0.11 ± 0.22	0.22 ± 0.22^∗^	0.09 ± 0.07	4.61 ± 1.46^∗∗∗^	0.76 ± 0.50^∗^	0.43 ± 0.19
Dentate gyrus	0.21 ± 0.13	2.62 ± 1.95^∗^	0.08 ± 0.09	10.57 ± 2.54^∗∗∗^	4.62 ± 0.76^∗^	0.59 ± 0.38

C: control group; F: formaldehyde-treated group; 200: 200 mg/kg MC-treated group; 500: 500 mg/kg MC-treated group; F200: formaldehyde plus 200 mg/kg MC-treated group; F500: formaldehyde plus 500 mg/kg MC-treated group. ^∗^*P* < 0.05 F200 group versus F group; ^∗∗^*P* < 0.001 C, MC200, MC500, and F500 groups versus F group.

## Data Availability

The data used to support the findings of this study are available from the corresponding author upon request.
